# Janus Kinase 2 Polymorphisms Are Associated with Risk in Patients with Gastric Cancer in a Chinese Population

**DOI:** 10.1371/journal.pone.0064628

**Published:** 2013-05-24

**Authors:** Li Yang, Dongxiao Liu, Song Liang, Renhua Guo, Zhihong Zhang, Hao Xu, Chao Yang, Yi Zhu

**Affiliations:** 1 Department of General Surgery, The First Affiliated Hospital of Nanjing Medical University, Nanjing, China; 2 Department of General Surgery, the Second Affiliated Hospital of South-east University, The Second Hospital of Nanjing, Nanjing, China; 3 Department of Medical Oncology, The First Affiliated Hospital of Nanjing Medical University, Nanjing, China; 4 Department of Medical Pathology, The First Affiliated Hospital of Nanjing Medical University, Nanjing, China; 5 Jiangsu Province Academy of Clinical Medicine, Institute of Tumor Biology, Nanjing, China; Sapporo Medical University, Japan

## Abstract

**Aim:**

To evaluate the impact of the Janus kinase 2 single nucleotide polymorphisms (SNPs) on gastric cancer risk.

**Methods:**

In this hospital-based, case–control study, the genotypes were identified by polymerase chain reaction–restriction fragment length polymorphism protocols in 661 individuals (359 gastric cancer patients and 302 age and sex matched cancer-free controls).

**Results:**

Both the frequency of A allele in rs2230724 and G allele in rs1887427 were more frequent in patients with gastric cancer (*P = *0.013 and 0.001, respectively). Compared with the common genotype, subjects with the (AG+AA) genotypes of rs2230724 and the (AG+GG) genotypes of rs1887427 had a 59% and 98% increased risk of developing gastric cancer, respectively (*P* = 0.010, adjusted OR = 1.59, 95% CI = 1.12–2.27; *P*<0.001, adjusted OR = 1.98, 95% CI = 1.39–2.81, respectively). Further stratified analysis showed that the association between the risk of gastric cancer and the rare genotypes of rs2230724 were more profound in the subgroups of elder individuals (>56 years), males, nonsmokers and urban subjects, while the association between the risk and the rare genotypes of rs1887427 persisted in subgroups of younger individuals (≤56 years), males, nonsmokers and both of rural and urban subjects.

**Conclusion:**

The JAK2 gene rs2230724 and rs1887427 polymorphisms are associated with an increased risk of gastric cancer in a Chinese Han population.

## Introduction

Gastric cancer remains the second leading cause of cancer-related mortality worldwide [1], [2]. It is widely accepted that gastric carcinogenesis is a complex, multistep and multifactorial process involving genetic and epigenetic alteration of protein-coding protooncogenes and tumor-suppressor genes [3]. Genetic polymorphisms have been considered as the main genetic elements in the development of various diseases, including gastric cancer. Although the precise molecular mechanism remains unclear, genetic polymorphisms are thought to play important roles in gastric carcinogenesis [4]. Moreover, our previous epidemiologic studies also provided the evidence that the risk of gastric cancer was associated with genetic polymorphisms [5], [6], [7], [8].

The Janus kinase 2 (JAK2) is a member of the family of tyrosine kinases (TKs) involved in cytokine receptor signaling. It is a key component of Janus kinase (JAK)/signal transducer and activator of transcription (STAT) signaling [9]. It has been shown to participate in multifarious crucial biological responses related to diverse processes during embryogenesis, cell proliferation, cell survival and carcinogenesis [9], [10], [11], [12]. The JAK/STAT pathway appears to be active in many solid tumors, including ovarian cancer, breast cancer, prostate cancer, lung cancer, gastric cancer as well as in hematologic malignancies [13], [14], [15], [16], [17], [18]. In addition, JAK2/STAT3 pathway is closely associated with epithelial mesenchymal transition (EMT) and tumor metastasis in colon cancer and ovarian cancer [18], [19]. Zhou *et al.* also found that the activation of JAK2 signaling pathway was likely to be associated with Helicobacter pylori-cytotoxin-associated protein A (CagA) induced high expression of gastrin in gastric cancer cells, which might be a main cause of stomach carcinogenesis [13]. Furthermore, it was reported that JAK2 was an oncogene and down-regulating the expression of JAK2 could significantly suppress the proliferation of gastric cancer cells [20].

The gene for JAK2 is located on chromosomal region 9p24.1. To date, several JAK2 single nucleotide polymorphisms (SNPs) have been identified that they were significantly associated with polycythemia vera (PV) and essential thrombocythemia (ET), including rs7046736, rs10815148, rs12342421, rs10758669, rs3808850 and rs10974947 [21]. Recently, JAK2 SNP rs10758669 was also reported to be associated with increasd susceptibility for Crohn’s disease (CD) [22], [23]. It is reported that SNPs in the coding region could affect the gene expression, while variants located in the promoter region of a gene predominantly affect the transcriptional activity and then the gene expression [24]. Among the SNPs, rs2230724 is a coding exonic SNP and rs1887427 is an upstream promoter’s SNP, and their change might play a role in the transcriptional activity and expression of JAK2 gene.

However, the role of JAK mutations in solid tumors is emerging, and it has been proposed that mutations identified so far may just be the tip of the iceberg [25]. Recently, a study reported that the JAK2 V617F mutation led to constitutive signaling through the JAK2 TK, resulting in increased cellular proliferation and resistance to apoptosis in hematopoietic cells [26]. Given the importance and the potential biological mechanism of rs2230724 and rs1887427, we propose that the JAK2 polymorphisms may be contribute to the differences in susceptibility and severity of gastric cancer. In this work, a hospital-based case-control study was conducted to examine the association between the JAK2 polymorphisms and the risk of development or progression of gastric cancer in a Chinese Han population.

## Materials and Methods

### Ethics Statement

This study was conducted in Jiangsu Province in east of China. Before the research was conducted, ethical board approval from the First Affiliated Hospital of Nanjing Medical University was obtained, and all of the subjects provided written informed consent.

### Subjects

This hospital-based case-control study comprised of 359 gastric cancer cases and 302 cancer-free controls. All cases were consecutively recruited at the First Affiliated Hospital of Nanjing Medical University between 2009 and 2010, and were diagnosed with gastric cancer based on histopathological evaluation. Those with secondary, recurrent tumors were excluded. All controls were randomly selected in the Department of General Surgery during the same period, without any history or diagnosis of malignancies and genetic disease. They were matched with the cases on age (±5 years) and sex. All of the subjects were unrelated Han nationality and from Jiangsu Province or its surrounding regions. The tumor histological grade was assessed according to World Health Organization criteria and was staged using the TNM staging of the International Union Against Cancer (UICC)/American Joint Committee on Cancer (AJCC) system (2002). All sample data, including age, gender, weight, residence, hypertension, diabetes, smoke, tumor location, histological grade, depth of tumor invasion, lymph node metastasis and clinical stage were obtained by questionnaire or from the clinical and pathologic records. Individuals who formerly or currently smoked ≥10 cigarettes per day for at least 2 years were defined as smokers.

### Genotyping

Genomic DNA was extracted from peripheral blood leukocytes by standard techniques. The protocol for genomic DNA extraction was described in our previous study [8]. A polymerase chain reaction (PCR)-restriction fragment length polymorphism (RFLP) assay was used to identify the JAK2 gene polymorphisms. The PCR was performed in a total volume of 20 µL reaction mixtures containing 2 µL 10× PCR buffer (MBI Fermentas), 1.75 mmol/L MgCl_2_, 0.15 mmol/L dNTP, 1 unit Taq polymerase (MBI fermentas), 150 ng genomic DNA and 0.25 µmol/L each primer (F-5′-TATTTGAGTTTCCCTGTATC-3′ and R-5′-CCTTGCCAAGTTGCTGTA-3′ for rs2230724; F-5′-TGTGGATGGGAAACCTAA-3′ and R-5′-AACTTCTACTCCTGCTTGG-3′ for rs1887427). For PCR amplification, after an initial denaturation at 95°C for 5 min, followed by 35 cycles of denaturation at 95°C for 30 s, annealing at 50°C for rs2230724 (54°C for rs1887427) for 30 s and elongation at 72°C for 60 s, with a final elongation at 72°C for 5 min.

**Figure 1:**
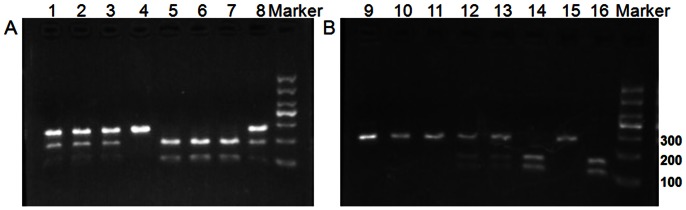
Digestion of PCR products by restriction enzymes. (A) Genotypes of rs2230724, Lanes 1–3 and 8 AG heterozygous (267, 183 and 84 bp); lane 4 AA homozygotes (267 bp); lanes 5–7 GG homozygotes (183 and 84 bp). (B) Genotypes of rs1887427, lanes 14 and 16 GG homozygotes (151 and 110 bp); lanes 9–11 and 15 AA homozygotes (261 bp); lanes 12 and 13 AG heterozygous (261, 151 and 110 bp).

**Figure 2:**
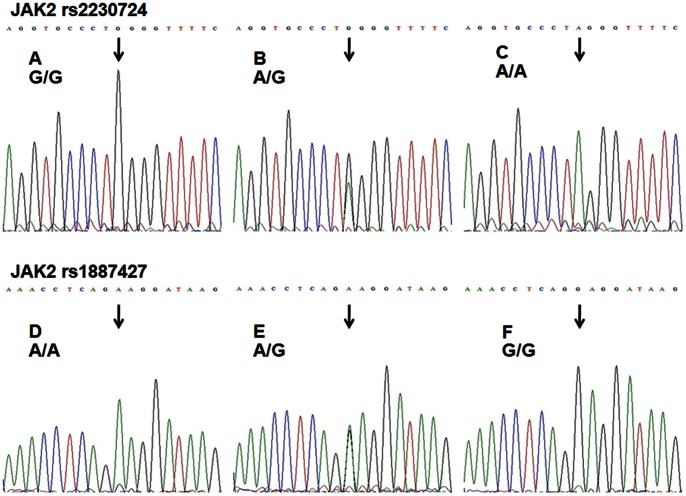
Direct sequencing results for the JAK2 gene rs2230724 and rs1887427 polymorphisms. The polymorphisms were detected by RFLP and confirmed by direct PCR sequencing. The single base directed with a black arrowhead was the SNP site. (A), (B) and (C) Representatives of GG, AG, AA genotypes of rs2230724 by direct DNA sequencing, respectively. (D), (E) and (F) Representatives of AA, AG, GG genotypes of rs1887427 by direct DNA sequencing, respectively.

For RFLP, The 267-bp and 261-bp PCR products of JAK2 polymorphisms (rs2230724 and rs1887427) were digested by the restriction enzyme BstNI and Bsu36I (New England BioLabs), 5 units for 16 h at 60°C and 37°C, respectively, followed by electrophoresis on a 3% agarose gel. For rs2230724, the common genotype homozygotes (GG) produced two bands at 183 and 84 bp, while the rare genotype homozygotes (AA) produced one band at 267 bp, and the heterozygous (AG) produced three bands at 267, 183 and 84 bp (Figure 1A). As for rs1887427, the common genotype homozygotes (AA) produced one band at 261 bp, while the rare genotype homozygotes (GG) produced two bands at 151 and 110 bp and the heterozygous (AG) produced three bands at 261, 151 and 110 bp (Figure 1B). About 10% of the samples were randomly selected to do the repeated assays for confirmation, and the results were 100% concordant. The genotypes of rs2230724 and rs1887427 were confirmed by direct sequencing (Figure 2). Two researchers, blinded to the clinical data, scored the genotypes independently.

### Statistical Analysis

All the analyses were carried out with the SPSS 13.0 (SPSS Inc., Chicago, IL, USA) and were based on two-tailed probability. Differences were considered statistically significant at *P*<0.05. Quantitative variables departing from the normal distribution were summarized as median and analyzed by Mann-Whitney rank sum test. Pearson’ χ2 test was used to test the difference in the distribution of categorical variables. The Hardy-Weinberg equilibrium of the JAK2 genotypes was evaluated by a goodness-of-fit χ2 test. Odds ratio (OR) and 95% confidence interval (95% CI) were calculated to evaluate the association between the polymorphism and the risk of gastric cancer. The crude OR was assessed using the Woolf approximation method and the adjusted OR was computed by unconditional logistic regression method with adjustment for age, sex, smoking status, residence, hypertension and diabetes.

## Results

### Demographic Information

A total of 661 subjects (359 cases and 302 controls) were analyzed in this study. Demographic characteristics of the study participants were shown in Table 1. The age and gender were well matched in the case and control groups. Moreover, there was no significant difference in weight, smoking status, residence, history of hypertension, and diabetes between the case and control group.

**Table 1:** Demographic information.

Characteristics	Cases (n = 359)	Controls (n = 302)	*P* value
Gender (male), n (%)	249 (69.4)	202(66.9)	0.497
Age (y)*	58 (49–67)	56 (49–65)	0.197
Weight (kg)*	61 (55–70)	63 (56–70)	0.129
Hypertension, n (%)	93 (25.9)	80 (26.5)	0.865
Diabetes, n (%)	29 (8.1)	35 (11.6)	0.128
Smoking, n (%)	85 (23.7)	62 (20.5)	0.332
Residence, n (%)			0.416
Rural	198 (55.2)	157 (52.0)	
Urban	161 (44.8)	145 (48.0)	

* Median (25^th^–75th percentiles).

### Distribution of JAK2 Genotype in Cases and Controls and Risk Estimates

For the polymorphisms of JAK2 (rs2230724 and rs1887427), the risks of carrying the rare genotypes were compared between the gastric cancer case and control groups, as shown in Table 2. There was no evidence for departure from Hardy-Weinberg equilibrium (HWE) in cases or controls (*P*>0.05 for all).

**Table 2:** Distributions of the JAK2 genotype in cases and controls and risk estimates.

JAK2 genotype	Cases N (%)	Controls N (%)	Crude OR (95% CI)	*P* value	Adjusted OR (95% CI)*	*P* value
overall	359	302				
rs2230724						
GG	78 (21.7)	91 (30.1)	1.00		1.00	
AG	188 (52.4)	149 (49.3)	**1.47 (1.02–2.13)**	**0.041**	**1.52 (1.04–2.21)**	**0.030**
AA	93 (25.9)	62 (20.5)	**1.75 (1.13–2.72)**	**0.013**	**1.79 (1.14–2.81)**	**0.011**
AG+AA	281 (78.3)	211 (69.9)	**1.55 (1.09–2.21)**	**0.014**	**1.59 (1.12–2.27)**	**0.010**
Allelic						
G	344 (47.9)	331 (54.8)	**1.32 (1.06–1.64)**	**0.013**		
A	374 (52.1)	273 (45.2)				
HWE	0.351	0.943				
rs1887427						
AA	229 (63.8)	232 (76.8)	1.00		1.00	
AG	117 (32.6)	62 (20.5)	**1.91 (1.34–2.74)**	**<0.001**	**2.01 (1.39–2.90)**	**<0.001**
GG	13 (3.6)	8 (2.6)	1.65 (0.67–4.05)	0.277	1.66 (0.66–4.18)	0.279
AG+GG	130 (36.2)	70 (23.2)	**1.88 (1.34–2.65)**	**<0.001**	**1.98 (1.39–2.81)**	**<0.001**
Allelic						
A	575 (80.1)	526 (87.1)	**1.68 (1.24–2.26)**	**0.001**		
G	143 (19.9)	78 (12.9)				
HWE	0.682	0.129				

The bold in the table indicates statistically significant data.

* Adjusted for age, sex, smoking status, residence, hypertension, and diabetes.

To our data, there were significant differences of allele and genotype frequency between gastric cancer patients and cancer-free controls. As to the rs2230724, the A allele frequency was significantly higher in the case group (52.1%; *P* = 0.013, OR = 1.32, 95% CI = 1.06–1.64) than in the control group (45.2%). With the GG genotype as reference, the genotypes (AG+AA) were associated with an increased risk of gastric cancer (*P* = 0.010, adjusted OR = 1.59, 95% CI = 1.12–2.27) after adjustment for age, sex, smoking status, residence, hypertension, and diabetes. Moreover, the AG heterozygotes had a 52% increased risk of gastric cancer (*P* = 0.030, adjusted OR = 1.52, 95% CI = 1.04–2.21), and the AA homozygotes had a 79% increased risk (*P* = 0.011, adjusted OR = 1.79, 95% CI = 1.14–2.81).

Considering the JAK2 polymorphism rs1887427, individuals with G allele had a 68% increased risk of gastric cancer (*P* = 0.001, adjusted OR = 1.68, 95% CI = 1.24–2.26). In addition, compared with the AA genotype, a significant association existed between (AG and GG) genotypes and patients with gastric cancer adjusted for age, sex, smoking status, residence, hypertension and diabetes (*P*<0.001, adjusted OR = 1.98, 95% CI = 1.39–2.81). Moreover, individuals with the AG heterozygotes were also increased in the patients compared with the gastric cancer-free controls (*P*<0.001, adjusted OR = 2.01, 95% CI = 1.39–2.90).

### Stratified Analysis of Polymorphism and Gastric Cancer Risk

As shown in Table 3, the results of stratified analyses by the median age of controls (56 years), sex, smoking status and residence with the JAK2 variant genotypes were performed. Considering the JAK2 polymorphism rs2230724, the increased risk of gastric cancer associated with the rare genotypes was significant in subjects ages >56 years (*P* = 0.002, adjusted OR = 2.25, 95% CI = 1.36–3.71), but not in subjects ages ≤56 years. In addition, the rare genotypes were associated with a 59% increased risk of gastric cancer in males subjects (*P = *0.036, adjusted OR = 1.59, 95% CI = 1.03–2.45), whereas the association was not statistically significant in females subjects. Stratification by smoking status revealed a significant association of the polymorphism with an elevated gastric cancer risk for nonsmokers (*P* = 0.004, adjusted OR = 1.81, 95% CI = 1.21–2.72), but not in smokers. In urban subjects, the rare genotypes were associated with a 143% increased risk of gastric cancer (*P = *0.001, adjusted OR = 2.43, 95% CI = 1.42–4.17), whereas the association was not statistically significant in rural subjects.

**Table 3:** Stratified analyses for variant *JAK2* genotypes in cases and controls.

Variable	(AG+AA)/GG for rs2230724		Allelic odds ratios and 95%confidence intervals for rs2230724		(AG+GG)/AA for rs1887427		Allelic odds ratios and 95%confidence intervals for rs1887427	
	Cases, n (%)	Controls, n (%)	Adjusted OR (95% CI)*	*P* value	Cases, n (%)	Controls, n (%)	Adjusted OR (95% CI)*	*P* value
Age (y), median								
≤56	127(35.4)/37(10.3)	118(39.1)/39(12.9)	1.11 (0.66–1.87)	0.693	76(21.2)/88(24.5)	43(14.2)/114(37.7)	**2.43 (1.51–3.91)**	**<0.001**
>56	154(42.9)/41(11.4)	93(30.8)/52(17.2)	**2.25 (1.36–3.71)**	**0.002**	54(15.0)/141(39.3)	27(8.9)/118(39.1)	1.60 (0.94–2.73)	0.083
Sex								
Females	88(24.5)/22(6.1)	71(23.5)/29(9.6)	1.69 (0.87–3.27)	0.121	39(10.9)/71(19.8)	26(8.6)/74(24.5)	1.68 (0.91–3.12)	0.099
Males	193(53.8)/56(15.6)	140(46.4)/62(20.5)	**1.59 (1.03–2.45)**	**0.036**	91(25.3)/158(44.0)	44(14.6)/158(52.3)	**2.18 (1.40–3.39)**	**0.001**
Smoking Status								
Smokers	62(17.3)/23(6.4)	46(15.2)/16(5.3)	1.03 (0.47–2.26)	0.948	24(6.7)/61(17.0)	17(5.6)/45(14.9)	1.15 (0.53–2.52)	0.727
Nonsmokers	219(61.0)/55(15.3)	165(54.6)/75(24.8)	**1.81 (1.21–2.72)**	**0.004**	106(29.5)/168(46.8)	53(17.5)/187(61.9)	**2.37 (1.59–3.55)**	**<0.001**
Residence								
Rural	150(41.8)/48(13.4)	116(38.4)/41(13.6)	1.08 (0.66–1.77)	0.752	73(20.3)/125(34.8)	43(14.2)/114(37.7)	**1.64 (1.02–2.62)**	**0.041**
Urban	131(36.5)/30(8.4)	95(31.5)/50(16.6)	**2.43 (1.42–4.17)**	**0.001**	57(15.9)/104(29.0)	27(8.9)/118(39.1)	**2.68 (1.55–4.64)**	**<0.001**

The bold in the table indicates statistically significant data.

* Adjusted for age, sex, smoking status, residence, hypertension, and diabetes.

The JAK2 polymorphism rs1887427 showed that a significantly elevated risk associated with the rare genotypes was found for younger subjects (ages ≤56 years; *P*<0.001, adjusted OR = 2.43, 95% CI = 1.51–3.91) but not in older subjects (ages >56 years). In male subjects, the rare genotypes were associated with a 118% increased risk of gastric cancer (*P = *0.001, adjusted OR = 2.18, 95% CI = 1.40–3.39), whereas the association was not statistically significant in female subjects. Stratification by smoking status revealed a significant association of the polymorphism with an elevated gastric cancer risk for nonsmokers (*P*<0.001, adjusted OR = 2.37, 95% CI = 1.59–3.55), but not in smokers. What`'s more, the rare genotypes were associated with an increased risk of gastric cancer both in urban and rural subjects (*P*<0.001, adjusted OR = 2.68, 95% CI = 1.55–4.64 and *P = *0.041, adjusted OR = 1.64, 95% CI = 1.02–2.62, respectively).

We also analyzed the associations between the JAK2 rare genotypes and clinicopathologic features of gastric cancer patients in our study (Table 4). However, no significant correlation was observed with the clinicopathologic variables.

**Table 4:** Associations between variant *JAK2* genotypes and clinicopathologic characteristics of gastric cancer.

Variable	(AG+AA) and GGfor rs2230724		Allelic odds ratios and 95%confidence intervals for rs2230724		(AG+GG) and AAfor rs1887427		Allelic odds ratios and 95%confidence intervals for rs1887427	
	AG+AA, N	GG, N	Adjusted OR (95% CI)*	*P* value	AG+GG, N	AA, N	Adjusted OR (95% CI)*	*P* value
Tumor differentiation								
Well	28	5	1.00		10	23	1.00	
Moderate	149	47	0.54 (0.19–1.57)	0.259	59	137	0.85 (0.36–1.99)	0.702
Poor	104	26	0.83 (0.27–2.55)	0.750	61	69	1.59 (0.66–3.83)	0.300
Depth of tumor infiltration								
T1	29	5	1.00		11	23	1.00	
T2	29	11	0.56 (0.16–1.91)	0.353	9	31	0.63 (0.20–1.93)	0.415
T3	78	20	0.55 (0.18–1.70)	0.299	38	60	1.19 (0.50–2.86)	0.694
T4	145	42	0.68 (0.24–1.96)	0.476	72	115	1.62 (0.71–3.67)	0.249
Lymph node metastasis								
Negative	91	19	1.00		43	67	1.00	
Positive	190	59	0.64 (036–1.15)	0.135	87	162	0.83 (0.52–1.35)	0.457
Localization								
Cardia	88	27	1.00		35	80	1.00	
Noncardia	193	51	1.11 (0.65–1.92)	0.699	95	149	1.48 (0.90–2.42)	0.121

* Adjusted for age, sex, smoking status, residence, hypertension, and diabetes.

## Discussion

JAK2 kinase is a member of the family of TKs involved in activation of several distinct intracellular signaling pathways. It is well known that JAK2 is a key component of Janus kinase (JAK)/signal transducer and activator of transcription (STAT) signaling [9]. JAKs can also stimulate the activity of kinase PI3K (phosphatidylinositol-3-kinase) of the PI3K/Akt/mTOR pathway which inhibits apoptosis and stimulates cellular proliferation [19], [26]. Several epidemiologic studies have investigated the association between the dysfunction of JAK2 and a variety of human diseases, including cancer [9], [13], [14], [15], [16], [17], [18], [27]. Importantly, Pham *et al*. [28]showed that the activation of JAK/STAT pathway participated in the tumorigenesis of gastric cancer. Ding *et al*. [20] also reported that down-regulating the expression of JAK2 could significantly suppress the proliferation of gastric cancer cells. Therefore, JAK2 might be crucial for the coordinated proliferation, differentiation and tumorigenesis of gastric cancer.

In our current hospital-based case–control study, we detected the effect of JAK2 rs2230724 and rs1887427 gene polymorphisms on gastric cancer. Compared with the common genotype, subjects with the (AG+AA) genotypes of rs2230724 and the (AG+GG) genotypes of rs1887427 had a 59% and 98% increased risk of developing gastric cancer, respectively. Further stratified analysis showed that the association between the risk of gastric cancer and the rare genotypes of rs2230724 were more profound in the subgroups of elder individuals (>56 years), males, nonsmokers and urban subjects, while the association between the risk and the rare genotypes of rs1887427 persisted in subgroups of younger individuals (≤56 years), males, nonsmokers and both of rural and urban subjects. According to our data, we for the first time found that the functional JAK2 polymorphisms conferred an increased risk of gastric cancer in a Chinese Han population.

Recently, great advances were made in the understanding of the pathogenesis of the polymorphisms of JAK2 in patients with various diseases, including hematologic malignancies and Crohn's disease [21], [22], [23], [29]. For example, rs2230724, a new SNP of JAK2, was associated with the susceptibility of acute leukemia and its subtypes. The A allele of rs2230724 was considered to be an important genetic determinant for acute leukemia and acute myeloid leukemia [29]. Moreover, Lee *et al.* [30] found that JAK2 SNP rs1887427 played major roles in prognosis and 13-cis-retinoic acid (13-cRA) response in patients with head and neck cancer. Patients with all 3 wild genotypes (JAK2 rs1887427 and two other gene polymorphisms) had a 76% reduction in second primary tumor (SPT)/recurrence following 13-cRA chemoprevention [30]. Nevertheless, to our knowledge, there is still no report on the association between JAK2 polymorphisms and the risk of gastric cancer. Based on these previous observations, this hospital-based case-control study was conducted to assess the role of JAK2 SNPs in gastric cancer. Our results showed that both the frequency of A allele in rs2230724 and G allele in rs1887427 were higher in patients with gastric cancer. The prevalence of A allele of rs2230724 was 45.2% in the control group, which was similar to another study in a Chinese population (44.0%) [29]. While the frequency of the G allele of rs1887427 was 12.9% in our control group, which was in the range of those in previous reports including the NCBI data (11.9% and 27.0% about Asian). It is probably that the study design, differences in sample size, selection of individuals and ethnic background can partly explain the inconsistence observed in this study and the others.

In the subgroup analyses, we found that JAK2 polymorphisms were associated with increased the risk of gastric cancer in males and nonsmokers. In general, gastric cancer is more common among men and smokers. A previous study has reported that noncardia cancer was more common in males than females by a ratio of approximately 2∶1 and gastric cardia cancer had a higher male-to-female ratio, of up to nearly 4.1∶1 in a Chinese population [31]. Our data suggest that JAK2 polymorphisms may play an important role in men with gastric cancer. However, the influence of these gene polymorphisms on gastric cancer was more critical in nonsmokers in this study. Cigarette smoke is confirmed to be an independent risk factor of gastric cancer [32]. The association between the polymorphism and gastric cancer risk could be masked by the overwhelming accumulated exposure to tobacco carcinogens in smokers so that the association is more evident in nonsmokers [7]. Further studies are required to detect if the JAK2 activity and its potential relevant cytokines are more prone to male and nonsmoker with gastric cancer.

As the age and residence are potential confounding factors for susceptibility of gastric cancer, we adjusted the variable of age and residence in the subgroup analyses. However, the association in the subgroup of age and residence with gastric cancer risk is inconsistent. Stratified results revealed that risk associated with the rare genotypes of rs2230724 was more profound in the subgroups of elder individuals (>56 years) and urban subjects; that the risk associated with the rare genotypes of rs1887427 persisted in subgroups of younger individuals (≤56 years) and both subgroups of rural and urban subjects. Therefore, further studies with larger sample size are required to verify the association between the polymorphisms and gastric cancer progression.

Our study has several limitations. Firstly, the sample size was relatively small, which may have led to weak statistical significance. In this study, although the prevalence of AG genotype of rs2230724 was not significantly different (52.4% *vs.* 49.3%, respectively) in the cohort of cases and negative cohort, the ratio of the prevalence of AG and GG (the reference) was significantly different (2.41 in cases *vs.* 1.64 in controls), which was also indicated by the low *P* value (*P* = 0.030) for the adjusted OR, suggesting an important role of JAK2 gene rs2230724 polymorphism in the risk of gastric cancer. Secondly, Helicobacter pylori is an independent risk factor for gastric cancer, but we did not explore the variable, because it was unethical to do the Helicobacter pylori test for every subject, especially for controls. Thirdly, in the subgroup analysis, we did not investigate the risk association with Lauren's classification, alcoholic drinking status and so on. Because all cases were consecutively recruited at the First Affiliated Hospital of Nanjing Medical University between 2009 and 2010 and we did not collect the clinical data of Lauren's classification, alcoholic drinking status *et al.* at that time. In our future study, we will definitely consider the related issues. Nevertheless, our results provided interesting information and valuable insights to future studies in this area.

In conclusion, our study for the first time demonstrates that the JAK2 gene rs2230724 and rs1887427 polymorphisms are associated with an increased risk of gastric cancer in the Chinese Han population. Further studies with larger sample size and in different population are required to verify our initial observations.
